# The effects of smoking and drinking on all-cause mortality in patients with dilated cardiomyopathy: a single-center cohort study

**DOI:** 10.1186/s40001-015-0171-z

**Published:** 2015-09-17

**Authors:** Xiaoping Li, Yang Liu, Rong Luo, Gang Li, Peng Luo, MingJiang Liu, Tao He, Wei Hua

**Affiliations:** Department of Cardiology, Sichuan Academy of Medical Sciences and Sichuan Provincial People’s Hospital, Chengdu, 610072 Sichuan People’s Republic of China; School of Medicine, University of Electronic Science and Technology of China, Chengdu, 610072 Sichuan People’s Republic of China; State Key Laboratory of Cardiovascular Disease, Cardiac Arrhythmia Center, Fuwai Hospital, National Center for Cardiovascular Diseases, Chinese Academy of Medical Sciences and Peking Union Medical College, Beijing, 100037 People’s Republic of China; Department of Cardiac Surgery, Beijing Anzhen Hospital, Capital Medical University, Beijing, 100029 People’s Republic of China; Key Laboratory of Thermoregulation and Inflammation of Sichuan Higher Education Institutes, Chengdu Medical College, Chengdu, 610500 People’s Republic of China; Cardiovascular Institute and Fuwai Hospital, Chinese Academy of Medical Sciences, Peking Union Medical College, Beijing, 100037 China; Hospital of the University of Electronic Science and Technology of China and Sichuan Provincial People’s Hospital, Chengdu, 610072 China

**Keywords:** Smoking, Drinking, Dilated cardiomyopathy, All-cause mortality, Survival

## Abstract

**Subject:**

Recent studies have shown that smoking and drinking are associated with poorer outcomes in patients with cardiomyopathy. The purpose of this study was to determine all-cause mortality in dilated cardiomyopathy (DCM) associated with smoking and drinking.

**Methods:**

An observational cohort study was undertaken in DCM patients from November 2003 to September 2011. A total of 1118 patients were enrolled, with a mean follow-up of 3.5 ± 2.3 years. Standard demographics were obtained, and transthoracic echocardiography and routine blood testing were performed shortly after admission. Outcome assessment was based on the all-cause death after admission.

**Results:**

The patients were divided into three groups: non-smokers (*n* = 593), mild-to-moderate smokers (*n* = 159) and heavy smokers (*n* = 366). The all-cause mortality rates showed no differences between the three groups (23.8, 20.8 and 24 %, respectively; log-rank *χ*^2^ = 1.281, *P* = 0.527). There was also no significant difference in mortality between non-drinkers (*n* = 747), mild drinkers (*n* = 142) and moderate drinkers (*n* = 229) (23.7, 23.2 and 22.3 %, respectively; log-rank *χ*^2^ = 2.343, *P* = 0.310). In the Cox analysis, neither the smoking (HR 0.971, *P* = 0.663) nor the drinking status (HR 0.891, *P* = 0.140) was a significant independent predictor of all-cause mortality in patients with DCM.

**Conclusion:**

In conclusion, there were no significant differences in mortality between the smoking- and drinking-related patient groups, indicating no effect of smoking and drinking on all-cause mortality in patients with DCM in the present large-scale study.

## Background

Dilated cardiomyopathy (DCM), a disease of the heart muscle characterized by ventricular dilatation and impaired systolic function, is the third most common cause of heart failure [1, 2]. The prognosis of patients with DCM is poor, with approximately half of the patients dying within 5 years of diagnosis, and it is necessary for the physician to predict which clinical course an individual patient may follow [1, 2].

Studies in mice and humans have shown that alcohol is a direct myocardial toxin that causes ultrastructural damage. Heavy drinking has been associated with left ventricular dysfunction and DCM, referred to as alcoholic cardiomyopathy [3, 4]. According to most studies, heavy drinking is associated with increased cardiovascular morbidity and mortality [5–7]. The US National Health and Nutrition Examination has stated that alcohol consumption has a linear relationship with mortality, with a slightly higher mortality risk for even light drinking [6, 7]. However, some studies have shown that moderate alcohol consumption has cardioprotective effects; reduces the risk of chronic heart failure, coronary artery disease and stroke; and decreases cardiovascular and all-cause mortality [8–12].

Cigarette smoking is a major modifiable risk factor for cardiovascular diseases, including coronary artery disease, stroke, peripheral vascular disease and congestive heart failure [13, 14]. Both smoking and exposure to passive smoke are major preventable causes of cardiovascular morbidity and mortality [15, 16]. Previous studies have indicated that smoking is related to cardiomyopathy [17] and is an important risk factor for idiopathic congestive cardiomyopathy [18]. However, recent studies have suggested that patients with DCM who smoke have a better prognosis than that of nonsmokers [19–21], although data from New Zealand show that smoking is associated with poorer survival in patients with DCM [22].

Although heavy drinking and smoking are known risk factors for cardiovascular mortality, their roles in DCM patients remain unclear [5, 19–22]. Therefore, in the present study, we aimed to evaluate the association of drinking and smoking with all-cause mortality in hospitalized patients with DCM in China.

## Subjects and methods

### Patients and follow-up

**Fig. 1:**
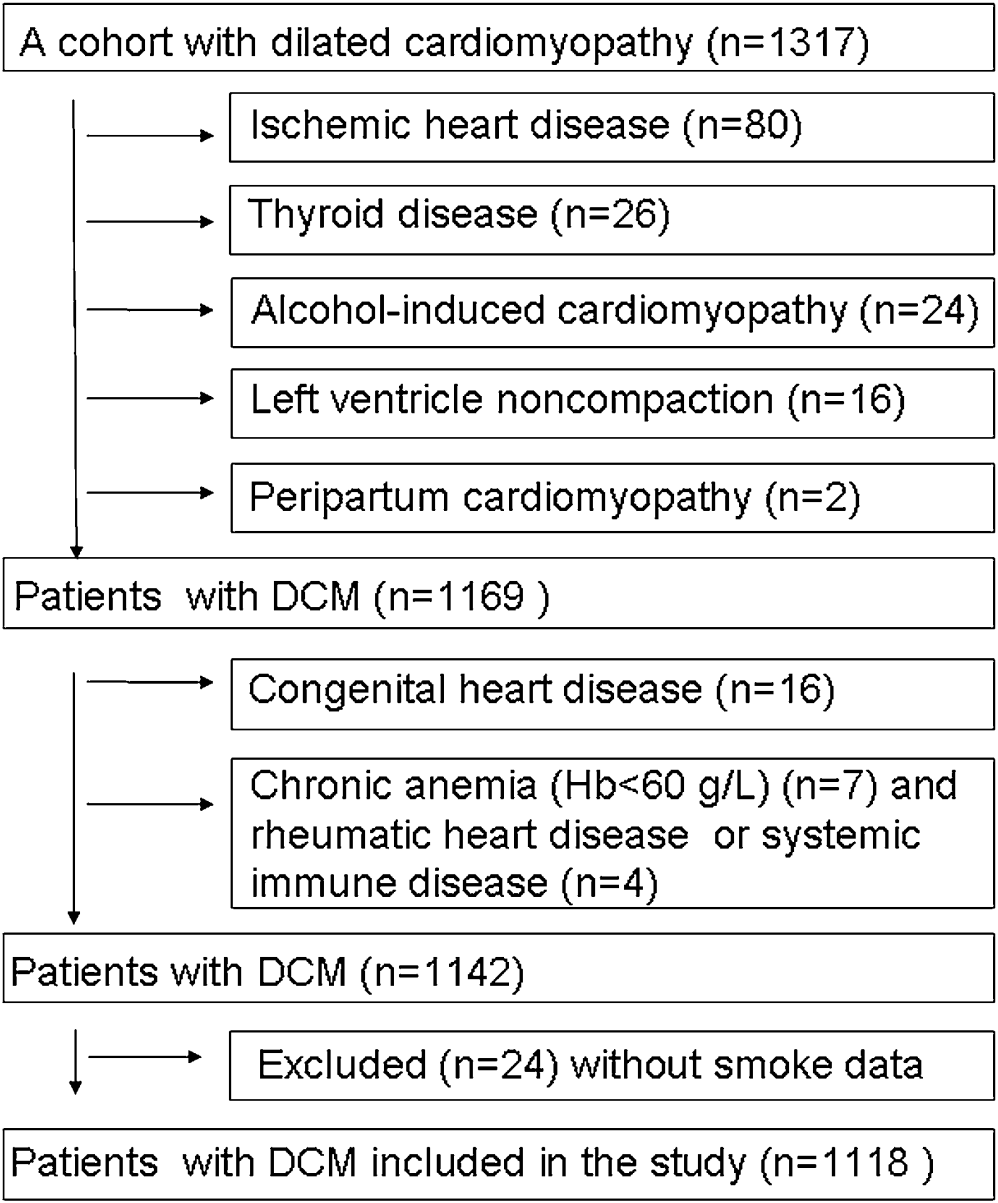
Flowchart for participants involved in the present study

A retrospective, observational cohort study of DCM patients was conducted from November 2003 to September 2011. The patients were admitted with symptoms of decompensation and physical signs of heart failure, and DCM was defined as systolic dysfunction (LVEF ≤50 %) with LV dilation in the absence of an apparent secondary cause of cardiomyopathy [23]. Of the 1317 enrolled patients, 175 patients were excluded from the study owing to the presence of various secondary cardiomyopathies, and data on smoking and drinking were lacking for 24 and 25 patients, respectively (Fig. 1). Thus, the final analysis included 1118 patients, with data on drinking lacking for 1 patient. The end point of the study was the all-cause mortality, which was assessed for all patients based on medical records and medical follow-up calls. Mortality data were obtained for all study patients from hospitalization to death. Data from patients who underwent cardiac transplantation were censored at the time of transplantation, if alive, to the date of the most recent clinical evaluation. The mean follow-up was 3.5 ± 2.3 years. The institutional review board approval was obtained.

### Smoking and drinking habits

The three categories of drinkers were: non-drinkers; mild drinkers, who reported drinking an average of one drink a day on the days on which they consumed alcohol during the previous year; and moderate drinkers, who reported drinking an average of two drinks a day on the days on which they consumed alcohol during the previous year (1 drink = 12 g alcohol) [24]. Three categories of smokers were considered: non-smokers, mild-to-moderate smokers, who had smoked less than 40-pack years (PY); and heavy smokers, who had smoked ≥40 PY [25]. PY are calculated as the number of cigarettes smoked per day multiplied by the years of smoking, divided by 20 [26].

### Echocardiography

Patients were imaged in the left lateral decubitus position using a commercially available system equipped with a 3.5 MHz transducer. Two-dimensional grayscale, pulsed, continuous and color Doppler data were acquired from the parasternal and apical views. For tissue Doppler imaging, the sector width was adjusted to obtain a frame rate of at least 115 frames/s. Left ventricular ejection fraction (LVEF) was calculated using Simpson’s biplane technique [27].

### Statistical analysis

The continuous variables are expressed as mean ± SD or as medians and interquartile ranges. The categorical variables between groups were compared using Chi-square tests. Hazard ratios with 95 % confidence intervals (95 % CIs) were used to estimate the adjusted relative risk for the various groups. Kaplan–Meier survival curves were compared using the log-rank test. Multivariate Cox proportional hazards regression models were applied to adjust for any confounding variables among groups. The analyses were conducted using SPSS (version 16.0, SPSS Inc., Chicago, IL, USA), and all tests were two sided. A *p* value <0.05 was used to determine statistical significance.

## Results

### Characteristics of the study population

The cohort consisted of 1118 patients with DCM: 300 (26.8 %) women and 818 (73.2 %) men, with a mean age of 51.2 ± 14.6 years. Among the 1118 patients, those of Han nationality were 1074 (96.1 %), while minor nationality were 44 (3.9 %); patients who lived in the north of the Yazi River were 970 (86.8 %) and those who lived in the south of the Yazi River were 148 (13.2 %). Of these subjects, 53.1 % (*n* = 593) were non-smokers, 14.2 % (*n* = 159) were mild-to-moderate smokers and 32.7 % (*n* = 366) were heavy smokers. In terms of drinking, 66.9 % (*n* = 747) of the cohort were non-drinkers; 12.7 % (*n* = 142) were mild drinkers and 20.5 % (*n* = 229) were moderate drinkers. Table 1 summarizes the baseline clinical characteristics of the cohort. Among the patients in the smoker and non-smoker groups, fewer women were smokers; a history of atrial fibrillation was more common among the heavy smokers; higher blood pressure and circulating creatinine levels and a greater *P* duration and left ventricle diameter were observed in the mild-to-moderate smokers and heavy smokers; and a larger right ventricle and left atrium diameter were observed in the heavy smokers. Among the patients in the non-drinker, mild-drinker and moderate-drinker categories, the number of women who were drinkers was lower, and a more frequent history of arterial hypertension, higher blood pressure levels and a larger left ventricle and left atrium diameter were observed in the moderate drinkers. There was no significant difference in terms of drug treatment at admission among the smokers or drinkers.

**Table 1:** Patient characteristics categorized by ventricular conduction blockage patterns

	All patients (*n* = 1118)	Non-smokers (*n* = 593)	Moderate smokers (*n* = 159)	Heavy smokers (*n* = 366)	*P* value	Non-drinkers (*n* = 747)	Mild drinkers (*n* = 142)	Moderate drinkers (*n* = 229)	*P* value
Age (years)	51.2 ± 14.6	51.0 ± 16.4	49.6 ± 13.9	52.1 ± 11.5	0.184	51.4 ± 15.8	50.4 ± 13.0	50.7 ± 11.2	0.650
Female gender, *n* (%)	300 (26.8)	280 (47.2)	13 (8.2)	7 (1.9)	<*0.001*	293 (39.2)	4 (2.8)	3 (1.3)	<*0.001*
History									
Disease duration (years)	2 (0.5–6)	3 (0.65–7)	2 (0.25–6)	3 (0.5–6)	0.167	2 (0.5–6)	2 (0.325–5)	3 (0.93–7)	*0.224*
Arterial hypertension, *n* (%)	300 (26.8)	142 (23.9)	48 (30.2)	110 (30.1)	0.068	178 (23.8)	41 (28.9)	81 (35.4)	*0.002*
Diabetes mellitus, *n* (%)	161 (14.4)	84 (14.2)	23 (14.5)	54 (14.8)	0.968	108 (14.5)	18 (12.7)	35 (15.3)	*0.783*
Stroke, *n* (%)	51 (4.6)	27 (4.6)	6 (3.8)	18 (4.9)	0.846	38 (5.1)	5 (3.5)	8 (3.5)	0.490
Atrial fibrillation, *n* (%)	260 (23.3)	125 (21.1)	32 (20.1)	103 (28.1)	0.025	165 (22.1)	32 (22.5)	63 (27.5)	0.231
Ventricular premature beat, *n* (%)	377 (33.7)	199 (33.6)	54 (34.0)	124 (33.9)	0.992	253 (33.9)	51 (35.9)	73 (31.9)	0.718
Ventricular tachycardia, *n* (%)	216 (19.3)	120 (20.2)	24 (15.1)	72 (19.7)	0.338	152 (20.3)	26 (18.3)	38 (16.6)	0.429
NYHA class III and IV, *n* (%)	821 (73.4)	439 (74.0)	106 (66.7)	276 (75.4)	0.102	558 (74.7)	99 (69.7)	164 (71.6)	0.367
Admission vital signs									
SBP (mm Hg)	113.1 ± 17.8	111.3 ± 17.4	115.0 ± 16.2	115.4 ± 18.7	*0.001*	112.0 ± 17.7	115.8 ± 17.4	115.1 ± 17.8	*0.012*
DBP (mm Hg)	72.5 ± 12.6	71.0 ± 12.3	75.2 ± 12.2	73.6 ± 13.0	<*0.001*	71.3 ± 12.6	74.2 ± 12.9	75.1 ± 12.2	<*0.001*
Heart rate, beats/min	80.8 ± 17.4	81.3 ± 17.8	79.9 ± 16.8	80.8 ± 17.4	0.611	81.1 ± 17.4	79.3 ± 17.4	80.9 ± 16.5	0.546
Laboratory values at admission									
AST (IU/L)	26 (20–35)	25 (19–34.75)	28 (20.75–36)	25 (20–35)	0.969	26 (19–35)	25 (20–34)	27 (21–37)	0.422
ALT (IU/L)	28 (19–45)	26 (18–43)	33 (22–58.5)	30 (20–45.5)	0.826	27 (19–44)	28 (20–45)	32 (22–47.75)	0.656
TB (mmol/L)	20.3 (15.1–30.625)	20.5 (15.1–31.5)	19 (15.175–28.525)	20.3 (15.1–29.5)	0.738	20.2 (15–30.775)	19.9 (16.2–29.35)	21 (14.85–30.3)	0.834
DB (mmol/L)	3.7 (2.5–6.575)	3.6 (2.4–6.9)	3.5 (2.5–5.775)	3.8 (2.6–6.25)	0.653	3.7 (2.4–6.8)	3.6 (2.5–6.2)	3.85 (2.5–6.575)	0.869
Glucose (mmol/L)	5.61 ± 1.83	5.61 ± 1.90	5.71 ± 2.04	5.58 ± 1.58	0.763	5.61 ± 1.83	5.48 ± 2.04	5.67 ± 1.56	*0.614*
TG (mmol/L)	1.56 ± 1.02	1.57 ± 1.06	1.60 ± 0.94	1.53 ± 0.98	0.736	1.54 ± 1.04	1.50 ± 0.81	1.67 ± 1.05	0.237
CHO (mmol/L)	4.60 ± 1.11	4.56 ± 1.12	4.61 ± 1.14	4.66 ± 1.10	0.458	4.58 ± 1.12	4.62 ± 1.05	4.67 ± 1.20	0.545
Creatinine (µmol/L)	92.8 ± 35.3	88.2 ± 34.7	98.2 ± 44.4	97.8 ± 30.6	<*0.001*	91.2 ± 38.2	94.7 ± 25.7	96.9 ± 29.9	0.082
BUN (µmol/L)	7.98 ± 3.99	7.79 ± 3.71	8.15 ± 5.62	8.20 ± 3.56	0.264	7.96 ± 4.06	7.63 ± 2.63	8.24 ± 4.45	*0.366*
Pro-NT BNP (fmol/ml)	1534.85 (790.725–2795.15)	1543.4 (828.85–2827.05)	1475.55 (701.05–2661.425)	1547.2 (790.35–2826.55)	0.353	1583 (825.3–2907.2)	1420 (721.8–2898.3)	1399.6 (737.3–2559.75)	0.149
Electrograph data									
QRS duration (ms)	119.7 ± 30.9	119.9 ± 32.4	119.7 ± 27.9	119.2 ± 29.9	0.941	119.6 ± 30.9	119.1 ± 31.0	120.0 ± 30.8	0.963
QT (ms)	405.8 ± 54.5	406.7 ± 55.7	406.9 ± 57.5	403.9 ± 51.1	0.727	406.4 ± 54.1	403.0 ± 47.9	404.8 ± 58.7	0.768
*P* (ms)	107.5 ± 21.7	105.0 ± 21.1	110.0 ± 20.6	110.7 ± 22.8	*0.002*	106.3 ± 21.8	108.2 ± 20.3	111.4 ± 21.8	*0.036*
PR (ms)	183.0 ± 33.0	183.0 ± 33.4	178.2 ± 32.6	185.2 ± 32.4	0.148	183.0 ± 33.8	182.5 ± 32.8	183.5 ± 30.6	0.965
Echocardiography data									
LVd (mm)	68.1 ± 9.4	67.3 ± 9.15	69.2 ± 9.10	69.1 ± 9.76	*0.004*	67.6 ± 9.27	69.6 ± 9.80	69.0 ± 9.40	*0.020*
LVEF (mm)	31.9 ± 8.4	31.5 ± 8.0	32.1 ± 8.9	32.3 ± 8.7	0.341	31.6 ± 8.2	32.1 ± 8.6	32.7 ± 9.0	0.232
RV (mm)	23.6 ± 5.4	23.3 ± 5.2	23.0 ± 4.7	24.5 ± 5.8	*0.004*	23.5 ± 5.4	23.9 ± 4.9	23.8 ± 5.7	0.583
LA (mm)	44.0 ± 7.7	43.2 ± 7.6	43.9 ± 7.9	45.4 ± 7.6	<*0.001*	43.6 ± 7.5	44.7 ± 8.6	45.1 ± 7.5	*0.020*
Medications at admission									
Diuretics, *n* (%)	1085 (94.6)	562 (94.8)	151 (95.0)	345 (94.3)	0.925	706 (94.5)	130 (91.5)	222 (96.9)	0.079
ACEI/ARB, *n* (%)	947 (84.7)	500 (84.3)	137 (86.2)	310 (84.7)	0.848	623 (83.4)	125 (88.0)	199 (86.9)	0.218
Beta-blockers, *n* (%)	1015 (90.8)	537 (90.6)	151 (95.0)	327 (89.3)	0.118	677 (90.6)	129 (90.8)	209 (91.3)	0.958
Digoxin, *n* (%)	898 (80.3)	486 (82.0)	124 (78.0)	288 (78.7)	0.593	587 (78.6)	118 (83.1)	193 (84.3)	0.111
Spironolactone, *n* (%)	1016 (90.9)	544 (91.7)	142 (89.3)	330 (90.2)	0.542	680 (91.0)	125 (88.0)	211 (92.1)	0.396

Data are expressed as mean ± SD, medians (interquartile range) or percentages; *P* values from independent-sample *t* tests are shown

Italics indicate *P* < 0.05

1 patient lacked drinking status data; 22 lacked electrocardiogram data; 43 lacked echocardiography data; 334 lacked NT-pro-BNP levels; 47 lacked fasting blood glucose levels; 29 lacked creatinine data; 40 lacked BUN levels; 83 lacked triglyceride data and total cholesterol levels; 71 lacked AST data; 72 lacked ALT data; 72 lacked TB data; 74 lacked DB data; and 17 lacked data on the medications at admission

*NYHA* New York Heart Association, *SBP* systolic blood pressure, *DBP* diastolic blood pressure, *AST* aspartate aminotransferase, *ALT* alanine aminotransferase, *BUN* blood urea nitrogen, *TG* triglyceride, *TC* total cholesterol, *BUN* blood urea nitrogen, *TB* total bilirubin, *DB* direct bilirubin, *BUN* blood urea nitrogen, *NT-pro-BNP* N-terminal fragment pro-brain natriuretic peptide, *LV* left ventricle, *LA* left atrium, *LVEF* left ventricular ejection fraction, *ACEI* angiotensin-converting enzyme inhibitor, *ARB* angiotensin receptor blocker

### Relationship between age, gender and all-cause mortality

**Fig. 2:**
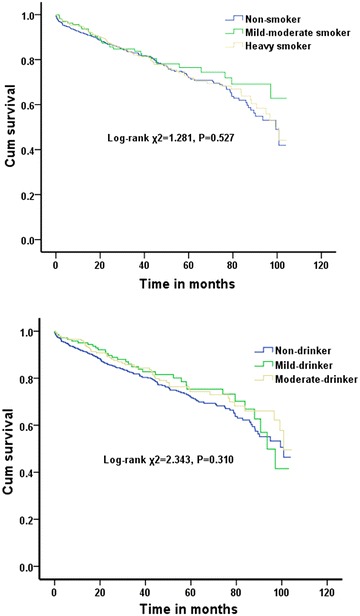
Kaplan–Meier survival curves for the smoking- and drinking-related groups of patients with dilated cardiomyopathy. The *upper panel* shows the survival curves among the non-smoker, mild-to-moderate-smoker and heavy-smoker groups (23.8, 20.8 and 24 %; log-rank *χ* ^2^ = 1.281, *P* = 0.527). The lower panel shows the survival curves among the non-drinker, mild-drinker and moderate-drinker groups (23.7, 23.2 and 22.3 %; log-rank *χ* ^2^ = 2.343, *P* = 0.310)

Of the 1118 patients studied, 262 (23.4 %) died and 3 (0.26 %) underwent heart transplantation during the mean follow-up of 3.5 ± 2.3 years. The all-cause mortality rates showed no difference between the non-smoker, mild-to-moderate-smoker and heavy-smoker groups (23.8, 20.8 and 24 %, respectively; log-rank *χ*^2^ = 1.281, *P* = 0.527). There was also no significant difference in mortality between the non-drinker, mild-drinker and moderate-drinker groups (23.7, 23.2 and 22.3 %, respectively; log-rank *χ*^2^ = 2.343, *P* = 0.310) (Fig. 2).

**Fig. 3:**
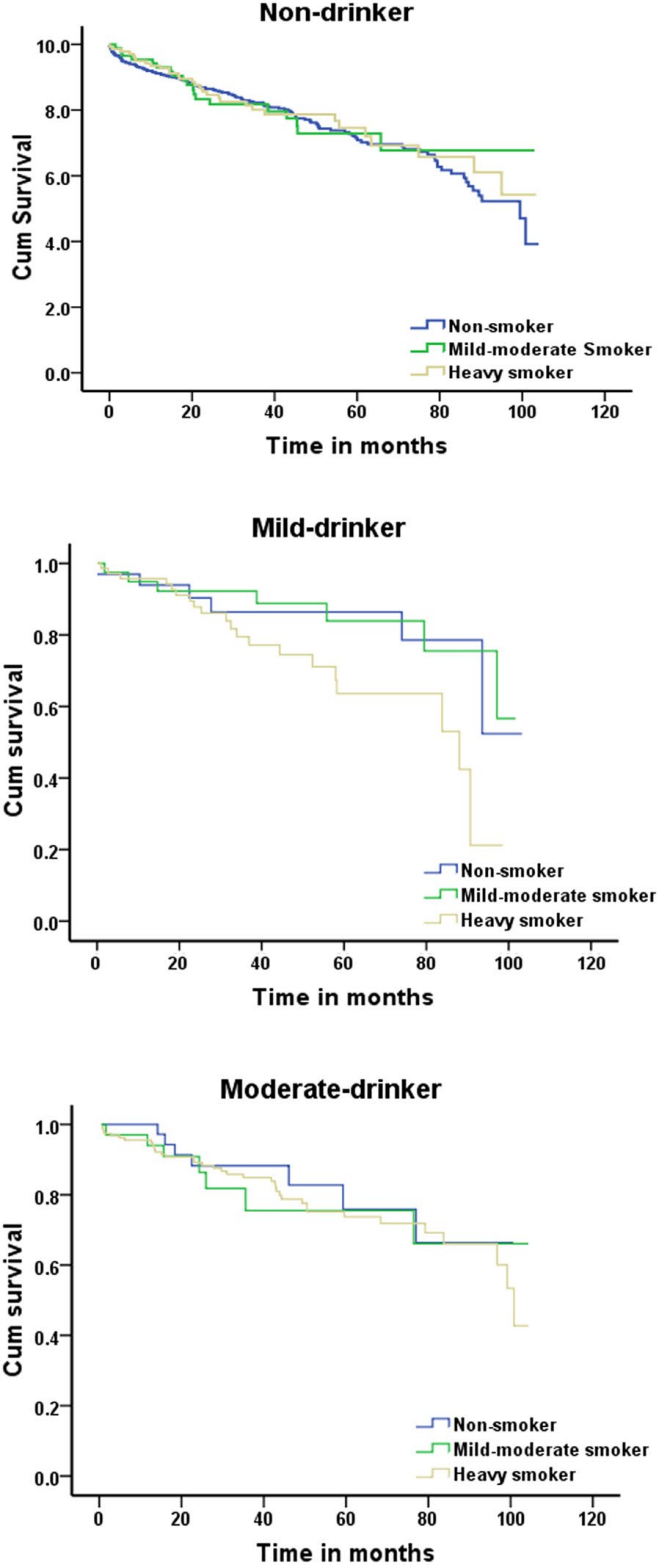
Kaplan–Meier survival curves for patients with DCM who were drinkers, stratified by their status as non-smokers, mild-to-moderate smokers and heavy smokers (log-rank *χ* ^2^ = 1.286, *P* = 0.526)

To determine whether smoking confers different degrees of risk between the different levels of drinkers with DCM, the entire cohort was divided into three subgroups and then further stratified according to the patients’ status as non-smokers, mild-to-moderate smokers and heavy smokers. In the non-drinking patients, the all-cause mortality rates for non-smokers, mild-to-moderate smokers and heavy smokers were 24.3, 22.1 and 22.5 %, respectively; among the mild drinkers, the all-cause mortality rates for non-smokers, mild-to-moderate smokers and heavy smokers were 18.2, 17.9 and 28.6 %, respectively; and in the moderate drinkers, the all-cause mortality rates for non-smokers, mild-to-moderate smokers and heavy smokers were 18.9, 20.6 and 23.4 %, respectively. There was no significant difference in all-cause mortality between the subgroups of patients with different drinking statuses according their different smoking statuses (log-rank *χ*^2^ = 1.286, *P* = 0.526) (Fig. 3).

### Cox proportional hazard models

When the clinical, laboratory, electrograph and electrocardiographic data were considered, univariate analysis revealed that age, history of hypertension, ventricular premature beat, New York Heart Association (NYHA) functional class, systolic blood pressure, diastolic blood pressure, *P* duration, QRS duration, left ventricle diameter, LVEF, right ventricle diameter, left atrium diameter, NT-pro-BNP, serum bilirubin, blood urea nitrogen, creatinine and fasting blood glucose were significant predictors of all-cause mortality in patients with DCM. Neither the smoking (HR 0.971, *P* = 0.663) nor the drinking status (HR 0.891, *P* = 0.140) was included in the Cox analysis. After adjustment for age, gender, smoking and drinking status, disease course, right ventricle and left atrium diameter, LVEF and serum creatinine, Cox multivariate analysis showed that ventricular premature beats, systolic blood pressure at admission, QRS duration, left atrium diameter and fasting blood glucose were powerful independent predictors of all-cause mortality in patients with DCM. Neither smoking nor drinking was found to be a predictor of all-cause mortality in the present study (Table 2).

**Table 2:** Cox regression of all-cause mortality in patients with DCM

Variable	Univariate analysis			Multivariate analysis		
	HR	95 % CI	*P* value I	HR	95 % CI	*P* value
Age	1.010	1.001–1.019	*0.024*	1.004	0.991–1.016	0.558
Sex	1.121	0.855–1.469	0.408	1.275	0.871–1.867	0.212
Ventricular premature beat	1.462	1.145–1.868	*0.002*	1.358	1.020–1.807	*0.036*
NYHA functional class	1.592	1.350–1.877	*<0.001*	1.223	0.990–1.511	0.062
Disease duration	1.028	1.011–1.044	*0.001*	1.012	0.990–1.035	0.291
Smoker	0.908	0.712–1.158	0.436	0.911	0.649–1.279	0.591
Drinker	0.817	0.630–1.060	0.129	0.951	0.669–1.352	0.779
Systolic blood pressure	0.982	0.975–0.989	*<0.001*	0.984	0.975–0.994	*0.001*
QRS duration	1.009	1.006–1.013	*<0.001*	1.010	1.005–1.014	*<0.001*
Left ventricle	1.037	1.025–1.050	*<0.001*	1.003	0.983–1.022	0.796
Right ventricle	1.061	1.037–1.086	*<0.001*	1.015	0.986–1.045	0.328
Left atrium	1.055	1.040–1.071	*<0.001*	1.047	1.025–1.069	*<0.001*
LVEF	0.963	0.948–0.978	*<0.001*	0.980	0.961–1.001	0.059
NT-pro-BNP	1.045	1.024–1.067	*<0.001*			
FBG	1.097	1.041–1.156	*<0.001*	1.097	1.026–1.173	*0.006*
Creatinine	1.004	1.002–1.007	*0.001*	1.003	1.000–1.006	0.053

The variables analyzed in the multivariate Cox mode included age, gender, ventricular premature beat, drinking and smoke status, disease duration, NYHA functional classes, systolic blood pressure, QRS duration, left ventricular, right ventriclular and left atrium diameter, LVEF, FBG and creatinine

Italics indicate *P* < 0.05

## Discussion

In this large-scale sample cohort study, we investigated the associations between smoking, drinking and all-cause mortality in patients with DCM. Our findings suggested that there was no predictive value of smoking and drinking for all-cause mortality in DCM patients; neither smoking nor drinking was an independent predictor of the all-cause mortality in patients with DCM.

Previous studies have found that that heavy drinkers exhibit a lower ejection fraction, greater end-diastolic volume, increased left atrial dimensions and increased left ventricular wall thickness, which occur in a dose-dependent fashion and precede the onset of clinical symptoms or physical findings [28, 29]. The mechanisms underlying alcohol-induced myocardial damage include cardiac myocyte apoptosis [30], alterations in the excitation–contraction coupling in cardiac myocytes [31], and increased oxidative stress [32] and activation of the renin–angiotensin system and the sympathetic nervous system [33]. However, moderate alcohol consumption has been proposed to confer protection against cardiovascular events while also increasing high-density lipoprotein cholesterol, decreasing platelet aggregation and coagulation, enhancing endothelial function, reducing inflammation, promoting antioxidant effects and decreasing the activity of angiotensin II (Ang II) [34–36]. In the present large-sample cohort study, although heavy drinkers were excluded owing to alcoholic cardiomyopathy, no significant differences were found between patients who were non-drinkers, mild drinkers and moderate drinkers. In a study in patients with a previous myocardial infarction, those who consumed small-to-moderate amounts of alcohol exhibited a lower total mortality [10]. However, the present study found no favorable effect of mild-to-moderate drinking on the all-cause mortality in the DCM patients.

Tobacco smoking has been solidly implicated in the etiology of cardiovascular diseases such as coronary artery disease, aortic aneurysm, stroke and peripheral vascular diseases [37], as cigarette smoking has been associated with higher serum levels of cholesterol, coronary vasomotor reactivity, platelet aggregation, and a prothrombotic state [38–41]. Additionally, an association between smoking and cardiomyopathy has been suggested by the results of several animal studies [42–44]. Possible mechanisms underlying the association between smoking and cardiomyopathy include direct damage to cardiac muscles (following damage to the myocardial mitochondria) and an increase in cardiac susceptibility to viral infections [43, 45]. Although it is well known that smoking increases cardiovascular morbidity and mortality [15, 16], the effect of smoking on the mortality of DCM patients cannot be conclusively determined based on the available data [19–22]. In the present study, there was no detectable influence of mild-to-moderate smoking and heavy smoking on the all-cause mortality in DCM patients. Although the all-cause mortality rate was lower among mild-to-moderate smokers than among non-smokers, this association did not achieve statistical significance.

## Conclusion

In conclusion, there were no significant differences in mortality between the smoking- and drinking-related patient groups, indicating no effect of smoking and drinking on all-cause mortality in patients with DCM in the present large-scale study.

## Limitations

The present study has several limitations. Like all hospital-based cohorts, the examined study population was a selected population of patients who had been referred for treatment. Because the NT-pro-BNP test was not commonly used until the later years of this study and its results were missing in 334 patients, we excluded NT-pro-BNP data from the multivariate Cox analysis to avoid the inclusion of potential confounding variables in the statistical analyses. Additionally, 14.1 % of the patients (*n* = 158) were lost to follow-up because of factors such as a lack of communication in rural areas; however, the main outcomes were not changed when we detected the losers in the Kaplan–Meier survival and Cox analyses. Ideally, all patients with DCM should be confirmed to be free of coronary artery disease. In practice, however, coronary arteriography is not routinely performed in all patients with congestive heart failure. Because retrospective studies cannot control the conditions under which patients are recruited or investigated, there was a very small subgroup of ischemic heart disease patients in the present study compared with the expected rates based on the numbers of patients who were subjected to coronary artery angiography, coronary CT scans or cardiac radionuclide imaging at other hospitals, as only 334 patients underwent coronary artery angiography and only 80 showed positive results at our hospital.

## Abbreviations

DCM: dilated cardiomyopathy
NYHA: New York Heart Association
SBP: systolic blood pressure
DBP: diastolic blood pressure
AST: aspartate aminotransferase
ALT: alanine aminotransferase
BUN: blood urea nitrogen
TG: triglyceride
TC: total cholesterol
BUN: blood urea nitrogen
TB: total bilirubin
DB: direct bilirubin
BUN: blood urea nitrogen
NT-pro-BNP: *N*-terminal fragment pro-brain natriuretic peptide
LV: left ventricle
LA: left atrium
LVEF: left ventricular ejection fraction
ACEI: angiotensin-converting enzyme inhibitor
ARB: angiotensin receptor blocker
CI: confidence interval
